# Gene expression profiles of wild-type and isoniazid-resistant strains of *Mycobacterium smegmatis*

**DOI:** 10.1016/j.dib.2015.05.006

**Published:** 2015-05-27

**Authors:** Jyothi Padiadpu, Priyanka Baloni, Nagasuma Chandra

**Affiliations:** a Department of Biochemistry, IISc, Bangalore 560012, India; b Supercomputer Education and Research Centre, IISc, Bangalore 560012, India; c Molecular Biophysics Unit, IISc, Bangalore 560012, India

## Abstract

The global variations in the gene expression pattern of drug treated (½X) and laboratory evolved drug-resistant strains (2XR and 4XR) of *Mycobacterium smegmatis* were obtained and compared with the *M. smegmatis* mc^2^ 155 (WT) strain. The genes exhibiting two-fold change and *p*-value <0.05 under the treated conditions have been considered as differentially expressed genes (DEGs). Overall, the numbers of DEGs observed are 1529 in ½X (596—up, 933—down), 1381 (899—up, 482—down) in 2XR and 716 in 4XR (267—up, 449—down) conditions. The data is publicly available through the GEO database with accession number GSE64132.

Specifications

**Table:** 

Organism/cell line/tissue	*Mycobacterium smegmatis*
Sex	NA
Sequencer or array type	*Mycobacterium smegmatis*, Agilent Custom *Mycobacterium smegmatis* Gene Expression 8×15 k Array (AMADID:020791) designed by Genotypic Technology Private Limited.
Data format	Raw and normalized microarray data
Experimental factors	Wild-type, (isoniazid) drug-treated, (isoniazid) drug-resistant strains
Experimental features	Expression profiling by array to identify variations in expression patterns due to isoniazid resistance
Consent	Level of consent allowed for reuse if applicable
Sample source location	NA

## 1 Direct link to deposited data

Deposited data can be found here

http://www.ncbi.nlm.nih.gov/geo/query/acc.cgi?acc=GSE64132.

## 2 Experimental design, materials and methods

### 2.1 Laboratory evolution of isoniazid-resistant strains of *M. smegmatis*

The *M. smegmatis* mc^2^ 155 was propagated in batch cultures in the presence of increasing concentrations of isoniazid (INH), using protocols similar in nature to that reported for the growth of drug-resistant strains of *Escherichia coli* [2,3]. Fresh isoniazid solutions were prepared from powder stocks every week and filter sterilized before use. Every 24 h, 0.8 ml of the culture (OD_600_~0.6) was inoculated to 100 ml fresh medium. This allows about 6–7 generations each day, and the process was continued for 4 days. The concentrations of isoniazid from a sub-inhibitory level to successively higher concentrations reaching up to four-fold MIC were administered sequentially. The culture was plated in Middlebrook 7H10 plates and colonies were observed. Those colonies growing on plates containing twice the inhibitory concentration of isoniazid were selected and subjected to increasing concentrations of the drug, in batch cultures. Subsequently, the colonies that grew at four times the estimated inhibitory concentration of isoniazid were selected. The strain thus obtained at four-fold MIC, was observed to grow even when subjected to nine times the minimal inhibitory concentration of isoniazid and is hereafter referred to as the ‘4XR’ strain. The two biological replicates are labeled as 4XR1 and 4XR2. A similarly generated strain, which was selected at two-fold MIC is referred to as ‘2XR’. Along with these the WT strain was subjected to sub-inhibitory concentration of isoniazid to observe the changes under drug stress which is hereafter referred to as ½X. These four different strains (WT, ½X, 2XR and 4XR) are compared in the study.

**Table:** 

**Strains**	**Description**
WT (Laboratory)	Maintained from the *M. smegmatis* mc^2^ 155.
½X	WT strain exposed to INH at a concentration of ½ MIC (MIC=10 µg/ml, so the cultures were exposed to 5 µg/ml for 6 h.
2XR	WT strain exposed to INH at a concentration 2 times MIC, (by gradually increasing INH from a concentration of 5 µg/ml of INH to 20 µg/ml over 4 days.
4XR	Further evolution of the 2XR strain by gradually exposing it to 4 times the MIC concentration of INH (starting from 2XR, i.e., 20 µg/ml, gradually increase to 40 µg/ml over a further period of 2 days). This strain was observed to grow even at 9 times the MIC of INH.

## 3 Transcriptome profiling

### 3.1 Strain and culture conditions

*M. smegmatis* mc^2^ 155 wild-type culture, ½X, 2XR, 4XR strains were grown in Middlebrook 7H9 media until 0.2–0.3 OD_600_ was observed. Once the required OD_600_ was reached, 20 ml of each culture was pelleted down and the supernatant was discarded. The pellets were resuspended in 100 µl of 1X PBS, snap-frozen in liquid nitrogen and stored at ^-^80 °C until RNA extraction was carried out.

### 3.2 RNA extraction and quality

RNA extraction from the samples was carried out using Qiagen׳s RNeasy minikit (Cat#74104). RNA concentration and purity was determined at an optical density ratio of 260/280 using the Nanodrop^®^ ND-1000 spectrophotometer (NanoDrop Technologies, Wilmington, DE) and the integrity of total RNA was verified on an Agilent 2100 Bioanalyzer using the RNA 6000 Nano LabChip (Agilent Technologies). The samples with OD_260_/_280_>1.8 and <2.2; OD_260_/_230_>0.5 and <2.4 were considered to be of optimal purity and considered for analysis.

### 3.3 Labeling and microarray hybridization

Labeling was effected using Agilent׳s Quick-Amp labeling Kit. In order to generate labeled complementary RNA, the random hexamer method of labeling was used followed by the T7 promoter based linear amplification. Hybridization was performed using Agilent׳s *In situ* Hybridization kit. Customized chips were used for microarray experiments of *M. smegmatis* mc^2^ 155 (8×15 k Array AMADID: 020791). The hybridization and the readings were carried out at Genotypic Technology, Bangalore, India.

### 3.4 Transcriptome data analysis

The raw signal intensity values were obtained for replicates of four different conditions namely WT, ½X, 2XR and 4XR. GeneSpring GX 12.6.1 software was used for carrying out intra- and inter-array normalization. We performed 75th percentile shift normalization and baseline transformation of the samples with respect to the control samples. Intra-array normalization deals with variability within a single array. In intra-array normalization, the gProcessed signal (dye normalized background subtracted signal intensity) is log 2 transformed and then for each of the array elements, the unique 75th percentile value is calculated. The log 2 transformed intensity values for each probe is subtracted by the calculated 75th percentile value of the respective array and expression values are obtained. Fold change values were calculated for genes in different conditions with respect to the median value of the WT samples. The student *t*-test was performed to calculate *p*-values.

### 3.5 Gene enrichment analysis

The DEGs were shortlisted and their corresponding Gene Ontology (GO) terms were identified using GORBI [5]. The unique GO terms were given as an input to REVIGO [6] for enrichment analysis.

## 4 Discussion

In the WT, 1597 genes were expressed (75th percentile) consistently in both replicates [1]. Number of *M. smegmatis* genes that were significantly differentially expressed (4-fold change) during treatment and drug-resistant conditions

**Table:** 

	½X	2XR	4XR

Total differentially expressed genes (DEGs)	397	328	89
Number of down regulated genes	226	98	61
Number of up regulated genes	171	230	28

The common up-regulated genes are enriched [5,6] in the functional; categories of *response to stress*, oxidation–reduction processes, lipid metabolism, ion transport, *response to stimulus*, molybdate transport and carbohydrate metabolism. The common down-regulated genes were enriched in processes such as amino sugar metabolism, mannose metabolism, reactive oxygen species metabolism, DNA-dependent transcription and detection of chemical stimulus. The transcriptome analysis revealed that there are many variations in the gene expression patterns in the drug-resistant strains. This indicates that drug resistance is associated with differential regulation of several genes in addition to the few genome sequence variations [4], leading to global variations.

## Conflict of interest

The authors declare that they have no conflict of interest.
